# Selective Ion Capturing via Carbon Nanotubes Charging

**DOI:** 10.1021/acs.analchem.2c00797

**Published:** 2022-05-17

**Authors:** Alexander Wiorek, Maria Cuartero, Gaston A. Crespo

**Affiliations:** Department of Chemistry, School of Engineering Science in Chemistry, Biochemistry and Health, KTH Royal Institute of Technology, SE-100 44 Stockholm, Sweden

## Abstract

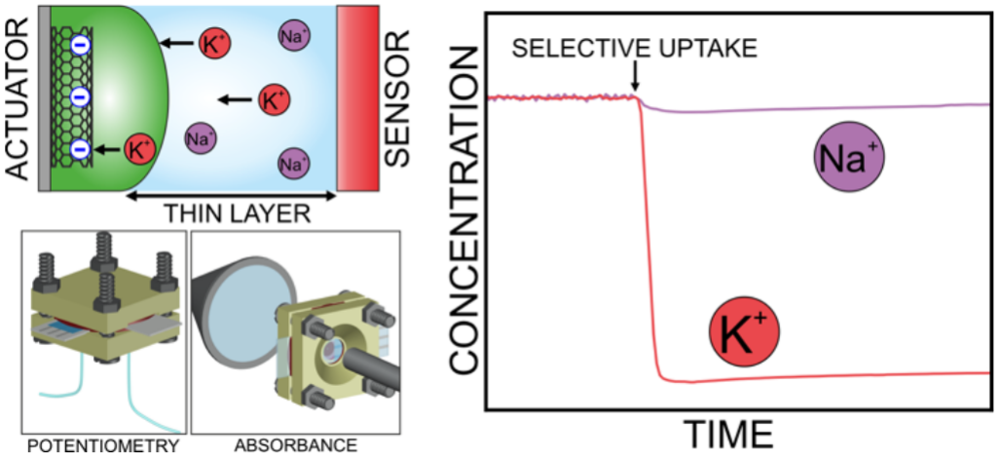

We present a phenomenon consisting of the synergistic effects of a capacitive material, such as carbon nanotubes (CNTs), and an ion-selective, thin-layer membrane. CNTs can trigger a charge disbalance and propagate this effect into a thin-layer membrane domain under mildly polarization conditions. With the exceptional selectivity and the fast establishment of new concentration profiles provided by the thin-layer membrane, a selective ion capture from the solution is expected, which is necessarily linked to the charge generation on the CNTs lattice. As a proof-of-concept, we investigated an arrangement based on a layer of CNTs modified with a nanometer-sized, potassium-selective membrane to conform an actuator that is in contact with a thin-layer aqueous solution (thickness of 50 μm). The potassium ion content was fixed in the solution (0.1–10 mM range), and the system was operated for 120 s at −400 mV (with respect to the open circuit potential). A 10-fold decrease from the initial potassium concentration in the thin-layer solution was detected through either a potentiometric potassium-selective sensor or an optode confronted to the actuator system. This work is significant, because it provides empirical evidence for interconnected charge transfer processes in CNT–membrane systems (actuators) that result in controlled ion uptake from the solution, which is monitored by a sensor. One potential application of this concept is the removal of ionic interferences in a sample by means of the actuator to enhance precision of analytical assessments of a charged or neutral target in the sample with the sensor.

Carbon nanotubes (CNTs) are among the most widely used ion-to-electron transducers in potentiometric sensors based on ion-selective membranes (ISMs). Here, the double layer capacitance of the CNTs is known to stabilize the inner interfacial potentials of the ISMs, which minimizes potential drifts in the potentiometric response.^1^ On the basis of a similar mechanism, in which a double layer is formed with the participation of ions in the adjacent phase, CNTs have also been utilized for the capacitive deionization of water.^2^ More specifically, differently functionalized CNTs have demonstrated the capability for reversible ion uptake from saline waters.^2,3^ By simply charging the CNTs through an applied potential, desalination of the sample by electrosorption of ions at the CNT–sample interface occurs.

Some of the CNT materials used for desalination have been classified as “ion specific”, in reference to the charge of the ion instead of the nature of the ion species.^3^ This kind of specificity is, indeed, exceeded by the selectivity of receptors traditionally used as ionophores in ISMs, which have not only charge specificity but are selective for only a single cation or anion species.^4^ Here, we aim to combine the fundamental principal of ion selectivity used in ISMs with the capacitive-based ion uptake concept employed in desalination technology. This CNT–ISM tandem is expected to provide a selective and controlled uptake of any ion in the sample, which could be particularly useful for removing/decreasing ionic interferences in a sample when performing analytical electrochemical or optical readouts. In addition, when the ion uptake process is demonstrated in a thin-layer sample (∼100 μm of thickness), a coulometric sensing approach is expected.

Figure 1a shows the mechanism for an actuator composed of an electrode modified with carboxylic acid-functionalized CNTs (COOH–CNTs, drop-casted layer), which, in turn, are covered with a spin-coated ISM (ca. 200 nm thick).^5^ The ISM is cation selective and contains an ionophore (L), a cation exchanger (Na^+^R_1_^–^), and a lipophilic salt (R_2_^+^R_3_^–^). Importantly, L is present in excess with respect to Na^+^R_1_^–^. The actuator is in contact with a thin-layer aqueous solution containing both the cation for which the membrane is selective (I^+^) and also other cations (J^+^). Initially, the system is composed of all unpolarized phases, and therefore, partitioning of ions among samples, ISMs, and CNTs is expected to be at equilibrium, representing a net zero change in concentration at either side of the sample–membrane or membrane–CNT interfaces. Notably, the Na^+^ in the membrane is replaced by I^+^ upon first contact with the solution in a superfast conditioning step (∼20 ms).^6^

**Figure 1:**
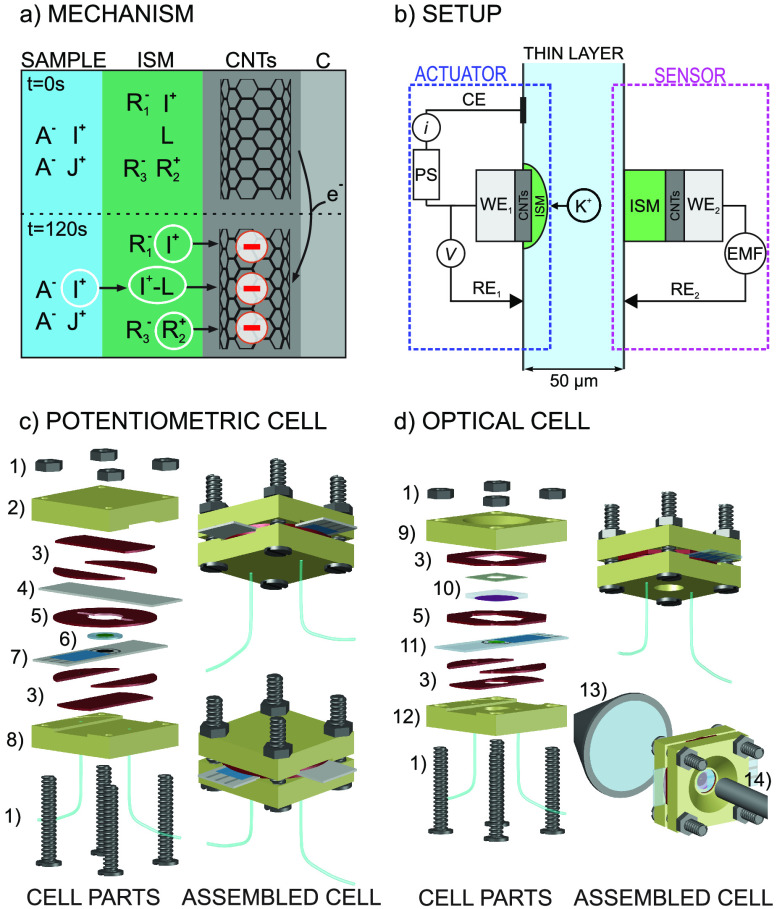
(a) Mechanism for ion uptake in the actuator. (b) Experimental setup where the sensor is potentiometric. (c) Microfluidic cell for the sensor and actuator system, where the sensor is potentiometric. (d) Microfluidic cell for the sensor and actuator system, where the sensor is an optode. ISM = ion-selective membrane; CNTs = carbon nanotubes; WE = working electrode; CE = counter electrode; RE = reference electrode; C = carbon; PS = power supply; EMF = electromotive force, compounds A^–^ = anion, I^+^ = cation, R_1_^–^ = anion of the cation exchanger, R_2_^+^,R_3_^–^ = lipophilic salt, L = ionophore, C = carbon electrode; (1) nuts and screws, (2) top electrode holder, (3) rubber and adhesive, (4) actuator, (5) 0.50 mm rubber spacer, (6) 450 μm mask with a membrane, (7) potentiometric sensor, (8) bottom electrode holder with inlet and outlet with corresponding tubings, (9) top electrode holder with a conical opening for the light path, (10) membrane optode, (11) transparent actuator, (12) bottom electrode holder with hole for light path (inlet and outlet), (13) light detector, and (14) light source.

The application of a negative potential step is expected to negatively charge the CNTs (i.e., increasing/changing the surface charge), and to comply with the electroneutrality condition, a net flux of the cation (I^+^) from the sample solution to the membrane is expected. This latter ion transfer is facilitated by the ionophore, being selective for only one cation (i.e., I^+^ but not J^+^). In essence, the ISM converts the CNTs into an ion-selective capturer via a capacitive mechanism, wherein only one ion species is taken up from the sample. In principle, any species with positive charge that is present in the membrane (i.e., R_2_^+^, I^+^ and/or IL^+^) could participate in the stabilization of the charged CNTs at the buried interface. One way to shed light on the relative engagement of each species would be with argon-based ion depth profiling coupled to synchrotron radiation-X-ray photoelectron spectroscopy (SR-XPS), which has demonstrated the necessary spatial resolution to determine concentration profiles in nanometer-sized membranes.^5^ However, such experiments are beyond the scope of this letter.

The strategy of tuning ion-transport processes across ISMs in backside contact with a chargeable ion-to-electron transducer has been applied through the use of different materials, such as CNTs, conducting polymers, and capacitive materials (mesoporous carbon), generating electroanalytical readouts that include voltammetry, chronoamperometry, and coulometry.^7−11^ In coulometry, charge transport of the ion from the sample to the solid contact has been suggested for obtaining the ion activity in the sample solution. To the best of our knowledge, none of the reported concepts have been applied together with thin-layer samples but rather in bulk solutions. Furthermore, the reported putative mechanism has not yet been confirmed by side in situ techniques capable of monitoring concentration changes in the solution owing to the ion-transfer process, a limitation addressed by the present work.

Figure 1b illustrates the setup for the concept proposed herein. In this setup, the sample is sandwiched between two working electrodes. One electrode behaves as the actuator (for controlled ion uptake), and the other behaves as a sensor (to monitor any change in ion concentration in the solution). The separation between electrodes is designed so as to provide a thin-layer domain for the sample (∼50 μm of thickness). As a proof-of-concept, the actuator is composed of a potassium-selective membrane (details provided in Supporting Information), and thus, selective potassium uptake from the sample solution is expected, which is to be followed by a potentiometric potassium-selective electrode confrontationally positioned.

Figure 1c illustrates each part of the microfluidic cell, configured to allocate the actuator–sample–sensor setup. In addition, top and bottom views of the assembled cell are provided. Both the actuator and sensor are realized as screen-printed electrodes modified with CNTs and ISMs for potassium, as described in the Supporting Information. The actuator is the working electrode of a three-electrode system (together with reference and counter electrodes), and it is activated by applying a constant potential of −400 mV (with respect to the open circuit potential, OCP) for 120 s by means of a potentiostat. The sensor works on the basis of potentiometry, which together with its corresponding reference electrode are connected to a potentiometer. Remarkably, the ion-uptake principle could be expanded to any other ion by tuning the membrane compositions, primarily by changing the ionophore. The rest of the parts of the cell ensure the appropriate positioning of the sensors, as well as the sample introduction and exchange in the thin-layer compartment. Importantly, the microfluidic cell may be slightly modified to integrate an optical sensor rather than a potentiometric one, as illustrated in Figure 1d (see also Figure S1, Supporting Information). Transparent substrates for the actuator and sensor, together with an adequate optical path, are necessary for such a purpose. In this letter, we used both potentiometric and optical readouts to confirm K^+^ uptake from the sample to the membrane.

First, we followed the K^+^ uptake in a 1 mM KCl solution, using the setup and cell with the potentiometric sensor (Figure 1b and c, respectively). The experiment was designed to monitor the signal before, during, and after the activation of the actuator through the application of a constant potential of −400 mV (respect to the OCP) for 120 s in 1 mM KCl solution. The potential displayed by the sensor (electromotive force, EMF) is converted into K^+^ concentration by means of a previous calibration graph (Figure S2). Under the described conditions, we compared the results provided by two different actuators: one with and one without the CNTs layer. As shown in Figure 2, the presence of CNTs in the actuator is necessary to observe a decrease in the K^+^ concentration in the thin-layer sample. Thus, as predicted in our hypothesis (Figure 1a), the charge disbalance promoting the ion transfer from the solution to the membrane is intrinsically generated by the CNTs, and this occurs only once the actuator is activated at a certain potential. Accordingly, the K^+^ concentration in the sample remained invariant until the application of the potential in the CNT-based actuator (after 50 s from experiment initiation). Then, it took ∼13 s for the K^+^ concentration to decrease from 1 to 0.1 mM (corresponding to a change in the initial potential of 60 mV), which then remained stable until the applied potential ceased. Finally, the K^+^ concentration tended to spontaneously increase up to the initial level because of the reversibility of the actuator. This process usually took approximately 15 min, across experimental replicates under identical conditions. Moreover, subsequent experiments performed after replacing the solution inside the microfluidic cell with fresh 1 mM KCl solution revealed that the K^+^ uptake process is, indeed, reproducible (Figure S3, Supporting Information), obtaining a final K^+^ concentration of 0.094 ± 0.016 mM (n = 3) in the sample.

**Figure 2:**
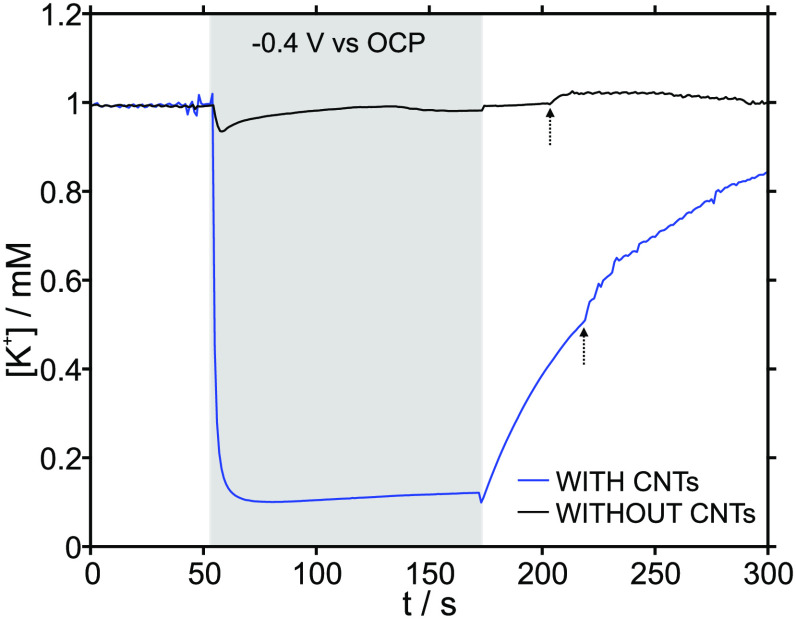
Concentration–time profiles for K^+^ in 1 mM KCl solution before, during, and after the activation of the actuator prepared with and without CNTs. The gray area in the plot represents the activation of the actuator (−0.4 V with respect to the OCP, for 120 s), and the arrows indicate activation of the peristaltic pump to regenerate the baseline for initiation of a new experiment.

Turning now to the time required for the sensor to report K^+^ uptake from the sample, it seems that the 13 s needed to observe the total decrease in K^+^ concentration (Figure 2) does not purely correspond to the K^+^ uptake *per se* but rather is likely affected by the response time of the sensor itself. The expected time for K^+^ depletion in the thin-layer sample can be theoretically estimated as follows. Briefly, Fick’s law is applied for the diffusion between equidistant elements in the thin layer sample (Figure S4a).^12^ Assuming that the kinetics for charge transfer across the sample–ISM interface is neglectable, the time for K^+^ depletion in the solution will be limited by the diffusion across the thin layer domain, which was estimated to be ca. 3 s, as detailed in the Supporting Information (Figure S4b and c). On the other hand, the response time (*t*_95_) for the sensor was between 3 and 8 s within the linear range of response (0.01–10 mM).

When the ion-selective membrane in the actuator did not contain any ionophore (composition provided in Table S1, Supporting Information), the potassium uptake was found to be only from 1 to 0.50 mM after the 120 s of activation of the actuator (black line in Figure S5). In the case of a membrane without the lipophilic salt (R_2_^+^R_3_^–^ = ETH500, Table S1), potassium uptake from 1 to 0.34 mM was observed (Figure S5, red line). These experiments confirmed that both the ionophore and the lipophilic salt are necessary to maximize K^+^ uptake. Impedance spectroscopy and cyclic voltammetry using membranes with ETH500 demonstrated a decrease in the membrane’s bulk resistance (Figures S6 and S7). Finally, the requirement for the cation exchanger (Na^+^R_1_^–^ = NaTFPB) was confirmed by comparison to a control membrane that did not contain this compound (composition provided in Table S1). In that experiment, the ion uptake was from 1 to 0.62 mM (Figure S5, blue line).

The selectivity of the ion-uptake process was further confirmed by a series of experiments in which the sample solution contained either 1 mM KCl, 1 mM NaCl, or 1 mM KCl/NaCl, and ion uptake was monitored by either a potassium- or sodium-selective electrode (detailed membrane compositions provided in Table S2). In a 1 mM KCl solution, K^+^ uptake was detected by the potassium sensor but not by the sodium sensor (Figure S8a). In a 1 mM NaCl solution, both the potassium and sodium sensors showed no significant change in the potential response, and thus, no ion uptake occurred (Figure S8b). In a 1 mM KCl + 1 mM NaCl solution, only the potassium sensor reported a change in the response, which was identical to the response detected in a pure 1 mM KCl solution (Figure S8c). We also performed control experiments in the presence and absence of O_2_ to rule out its involvement in the charging mechanism of CNTs by second side reactions (Figure S9).

An optical potassium sensor (a membrane-based optode; see Supporting Information) was also used to validate K^+^ uptake, using the microfluidic cell shown in Figure 1d. As with the potentiometric sensor, the optode was calibrated before the actuator–sensor experiments to convert the optical signal into dynamic K^+^ concentration in the thin-layer sample (Figure S10a). Notably, the change of the actuator substrate (from a carbon-based to a transparent one) required a different applied potential (−700 mV with respect to the OCP) because of the change in the OCP. In addition, a longer holding time (20 min) was applied, due to a slower response time of the optical sensor (*t*_95_ ranging from 300 to 520 s, Figure S10a). The optode showed that K^+^ concentration decreased from 1 to ca. 0.2 mM (Figure S10b), which is indeed very similar to that observed with the potentiometric sensor.

The efficiency of the potassium-uptake process, defined as the relative reduction in K^+^ concentration in the sample solution, was investigated at different KCl concentrations, ranging from 0.1 to 10 mM. Figure 3 depicts the dynamic concentration profiles obtained at different initial KCl concentrations before, during, and after applying −400 mV (with respect to the OCP) for 120 s in the actuator. In all cases, the uptake process caused an approximately 10-fold reduction from the initial K^+^ concentration. This fixed reduction, independent of the initial K^+^ concentration, is likely explained by electrostatic repulsion of the negative charges in the membrane, which could affect the rate of cation uptake in the membrane when the concentration is drastically lowered in the sample, thus limiting the final concentration decrease. Moreover, we investigated any possible relationship between the K^+^ concentration in the thin layer sample and the charge generation in the actuator. The generated charge (integration of the current profiles) showed a trend toward increasing K uptake (Figure S11). Interestingly, the charge exhibited a linear relationship to the log of the total concentration change within the range from 0.4 to 7 mM KCl (inset in Figure S11). While the reason for this logarithmic relationship is not yet clear, previous studies with conducting polymers have suggested a similar trend.^10^

**Figure 3:**
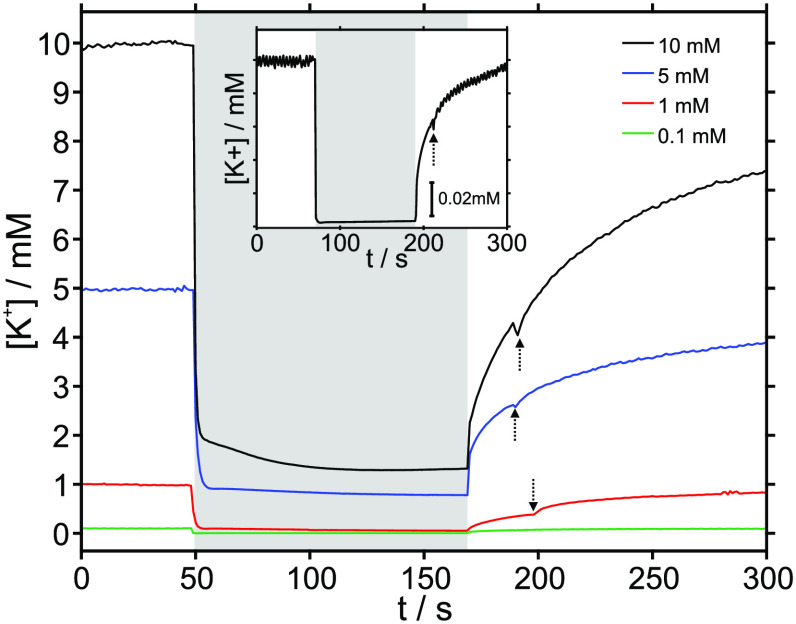
Concentration–time profiles observed in 0.1, 1, 5, and 10 mM KCl solution before, during, and after the activation of the actuator prepared with and without CNTs. The gray area in the plot represents the activation of the actuator (−0.4 V with respect to the OCP for 120s), and the arrows indicate activation of the pump to regenerate the baseline. Inset: magnification for the 0.1 mM KCl solution.

Finally, different types of CNTs were investigated as part of the actuator design, namely, COOH–CNTs, ODA-CNTs (octadodecylammonium-CNTs), and the unmodified CNTs (details are provided in the Supporting Information). Figure 4 presents the corresponding K^+^ concentration profiles. A larger uptake was observed in this order: ODA-CNTs < COOH–CNTs < CNTs (with corresponding changes from 1 mM KCl to 0.45, 0.1, and 0.02 mM, respectively). Additionally, impedance spectroscopy on the different types of CNTs (Figure S12) revealed a larger capacitance in the following order: COOH–CNTs < CNTs < ODA-CNTs (71, 2000, and 10000 μF/cm^2^, respectively), which is consistent with the literature,^13,14^ and suggests that other factors in addition to the capacitance itself can explain the uptake efficiency of the different CNTs.

**Figure 4:**
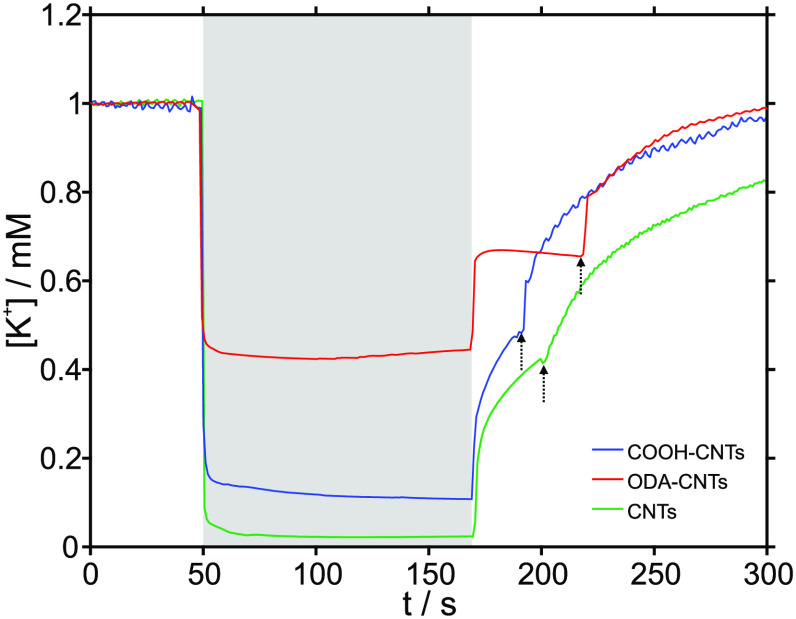
Concentration–time profiles for potassium in 1 mM KCl solution before, during, and after the activation of the actuator prepared with COOH–CNTs, ODA-CNTs, and unmodified CNTs. The sensor is a potentiometric one. The gray area in the plot represents the activation of the actuator (−0.4 V respect to the OCP for 120 s), and the arrows indicate when the peristaltic pump was turned on to regenerate the baseline.

We hypothesize that the order described above should be aligned with charge generation in the CNTs, together with its stabilization requirements, which could be dependent on the structural/packing differences between the CNTs. For example, steric impediments should more easily be found at the CNT–membrane interfaces when the substitution levels in the CNTs increase, while the lower degrees of sp^2^-hybridization would reduce the electronic conductances of the CNTs by lowering the charging capacities.^5^ This is likely why the unmodified CNTs exhibited a larger K^+^ uptake than the COOH–CNTs. Also, pi–pi interactions leading to pi–cation–pi bonding in the CNT could be a factor to be considered. Having demonstrated an effective K^+^ uptake, the observed differences could be further investigated with the help of additional techniques, such as XPS.

Overall, the potential of the actuator–sensor system for selective K^+^ uptake and its monitoring in thin-layer samples has been demonstrated. The concept is versatile enough for key changes in the design to permit the selective uptake of any cation or anion, as long as a selective receptor is available. This uptake offers an obvious application in the removal of interferences to facilitate analytical detection in a sample, which is to be achieved by the sensor (e.g., for electrochemical or optical readout). Further characterization of the system interfaces by means of synchrotron radiation techniques may help to confirm the working mechanism proposed herein.

## Supporting Information Available

Experimental section, diffusion profiles, membrane compositions, scheme of the optical sensor, calibrations, reproducibility, control experiments, impedance, and selectivity (PDF)

## References

[ref1] Crespo G. A.; Macho S.; Rius F. X. Ion-Selective Electrodes Using Carbon Nanotubes as Ion-to-Electron Transducers. Anal. Chem. 2008, 80 (4), 1316–1322. 10.1021/ac071156l. 18271511

[ref2] Li H.; Pan L.; Lu T.; Zhan Y.; Nie C.; Sun Z. A comparative study on electrosorptive behavior of carbon nanotubes and graphene for capacitive deionization. J. Electroanal. Chem. 2011, 653, 40–44. 10.1016/j.jelechem.2011.01.012.

[ref3] Yang J.; Zou L.; Choudhury N. R. Ion-selective carbon nanotube electrodes in capacitive deionisation. Electrochem. Acta 2013, 91, 11–19. 10.1016/j.electacta.2012.12.089.

[ref4] Cuartero M.; Crespo G. A. All-solid-state potentiometric sensors: A new wave for in situ aquatic research. Curr. Opin. Electrochem. 2018, 10, 98–106. 10.1016/j.coelec.2018.04.004.

[ref5] Cuartero M.; Bishop J.; Walker R.; Acres R. G.; Bakker E.; De Marco R.; Crespo G. A. Evidence of double layer/capacitive charging in carbon nanomaterial-based solid contact polymeric ion-selective electrodes. Chem. Commun. 2016, 52, 9703 10.1039/C6CC04876E. 27405722

[ref6] Yuan D.; Cuartero M.; Crespo G. A.; Bakker E. Voltammetric Thin-Layer Ionophore-Based Films: Part 2. Semi Empirical Treatment. Anal. Chem. 2017, 89, 595–602. 10.1021/acs.analchem.6b03355. 27976860

[ref7] Liu Y.; Wiorek A.; Crespo G. A.; Cuartero M. Spectroelectrochemical Evidence of Interconnected Charge and Ion Transfer in Ultrathin Membranes Modulated by a Redox Conducting Polymer. Anal. Chem. 2020, 92, 14085–14093. 10.1021/acs.analchem.0c03124. 32972129 PMC7584340

[ref8] Wang H.; Yuan B.; Yin T.; Qin W. Alternative coulometric signal readout based on solid-contact ion-selective electrode for detection of nitrate. Anal. Chim. Acta 2020, 1129, 136–142. 10.1016/j.aca.2020.07.019. 32891383

[ref9] Xu K.; Cuartero M.; Crespo G. A. Lowering the limit of detection of ion-selective membranes backside contacted with a film of poly(3-octylthiophene). Sens. Actuators B Chem. 2019, 297, 126781 10.1016/j.snb.2019.126781.

[ref10] Vanamo U.; Hupa E.; Yrjänä V.; Bobacka J. New Signal Readout Principle for Solid-Contact Ion-Selective Electrodes. Anal. Chem. 2016, 88, 4369–4374. 10.1021/acs.analchem.5b04800. 27018524

[ref11] Pergel E.; Gyurcsányi R. E.; Tóth K.; Lindner E. Picomolar Detection Limits with Current-Polarized Pb^2+^ Ion-Selective Membranes. Anal. Chem. 2001, 73, 4249–4253. 10.1021/ac010094a. 11569816

[ref12] Morf W. E.; Pretsch E.; De Rooij N. F. Computer simulation of ion-selective membrane electrodes and related systems by finite-difference procedures. J. Electroanal. Chem. 2007, 602 (1), 43–54. 10.1016/j.jelechem.2006.11.025. PMC2849319 20376294

[ref13] Wu Z.; Wang Z.; Yu F.; Thakkar M.; Mitra S. Variation in chemical, colloidal and electrochemical properties of carbon nanotubes with the degree of carboxylation. J. Nanoparticle Res. 2017, 19, 16 10.1007/s11051-016-3697-2. PMC5642976 29046611

[ref14] Yuan D.; Anthis A. H. C.; Ghahraman Afshar M.; Pankratova N.; Cuartero M.; Crespo G. A.; Bakker E. All-Solid-State Potentiometric Sensors with a Multiwalled Carbon Nanotube Inner Transducing Layer for Anion Detection in Environmental Samples. Anal. Chem. 2015, 87 (17), 8640–8645. 10.1021/acs.analchem.5b01941. 26272001

