# Supplementary material for: Selective Ion Capturing via Carbon Nanotubes Charging
Source: Anal Chem. 2022 May 17;94(21):7455–9. doi: 10.1021/acs.analchem.2c00797 (PMC9161223; doi:10.1021/acs.analchem.2c00797)
Supplement: Supplementary file 1 — ac2c00797_si_001.pdf [file ac2c00797_si_001.pdf]

Supporting Information for:

Selective Ion Capturing via Carbon Nanotubes Charging

Alexander Wiorek, Maria Cuartero*, and Gaston A. Crespo*

Department of Chemistry, School of Engineering Science in Chemistry, Biochemistry and Health, Royal Institute of Technology, KTH, SE-100 44 Stockholm, Sweden

Corresponding author (*): mariacb@kth.se; gacp@kth.se

Table of Contents

1. **Experimental Section** ... S-2
   - **Reagents, materials and instrumentation** ... S-2
   - **Preparation of the actuators.** ... S-2
   - **Preparation of the sensors.** ... S-3
2. **Additional Electrochemical Experiments** ... S-4
   - **Influence of ETH500** ... S-4
   - **Impedance on the different CNTs.** ... S-4
3. **Calculations of Diffusion Profiles in the Thin Layer** ... S-5
4. **Tables** ... S-6
   - **Table S1** ... S-6
   - **Table S2** ... S-6
5. **Figures** ... S-7
   - **Figure S1** ... S-7
   - **Figure S2** ... S-7
   - **Figure S3** ... S-8
   - **Figure S4** ... S-8
   - **Figure S5** ... S-9
   - **Figure S6** ... S-9
   - **Figure S7** ... S-10
   - **Figure S8** ... S-11
   - **Figure S9** ... S-12
   - **Figure S10** ... S-13
   - **Figure S11** ... S-14
   - **Figure S12** ... S-14

References ... S-15

1. Experimental Section

Reagents, materials and instrumentation. High molecular weight poly(vinyl chloride) (PVC), bis(2-ethylhexyl)sebacate (DOS), sodium tetrakis[3,5-bis-(trifluoromethyl)phenyl]borate (NaTFPB), potassium ionophore I (Valinomycin), tetradodecylammonium tetrakis(4-chlorophenyl)borate (ETH 500), tetrahydrofuran (> 99.9%, THF), tetrahydrofuran (THF), sodium ionophore X (4-tert-Butylcalix[4]arene-tetraacetic acid tetraethyl ester), potassium chloride (99.5%, KCl), sodium chloride (99.5%) and Trizma Base® (99.9%), were purchased from Sigma Aldrich. Hydrochloric acid (reagent grade, 1 M) was purchased from VWR chemicals and magnesium chloride (99%) was purchased from Alfa Ceasar. Multi-walled Carbon Nanotubes (outer diameter 30-50 nm, length 50 μm, purity>95 wt%, ash>1.5 wt%, specific surface area>60 m^2/g) was purchased from HeJi. All solutions were prepared with ultrapure water having resistance of 18.2 MΩ cm (Milli-Q water systems, Merck Millipore).

The carboxylated CNTs (COOH-CNTs) were synthesized as follows. 5 g of MWNTs were added into 200 mL concentrated HNO_3 and H_2SO_4 (1:3) and stirred for 24 h at room temperature. Finally, the COOH-CNTs was washed Milli-Q water, collected by filtration and finally completely dried in the oven at 60°C overnight.

The octadodecylamine-modified CNTs (ODA-CNTs) were synthesized as reported elsewhere.[1] 1 g of MWCNTs were first carboxylated under reflux in concentrated HNO_3 and H_2SO_4 (1:3) mixture for 1 hour at 100°C. They were subsequently washed with Milli-Q water and collected through filtering and completely dried in the oven over night at 60°C. The carboxylated CNTs were then added to 20 mL thionyl chloride and 1 mL dimethylformamide and refluxed over night at 70°C. Excess solvents were then removed from the CNTs by rotary evaporation, whereafter it was mixed with 1 g ODA and heated to 100°C for 96 h. The final product was first cooled to room temperature, whereafter unreacted ODA was removed by ultra-sonification in ethanol five times and finally allowed to dry before being put in storage for further usage.

The electrochemical experiments with the actuator were performed with a PGSTAT204 Autolab potentiostat (Metrohm Nordic AB) connected to a PC and operated with the Nova 2.1.4 software. Potentiometric data from the potentiometric sensors were recorded with a high input impedance ($10^{15}\Omega$) EMF16 multichannel data acquisition device, Lawson labs EMF16 Interface (Lawson Laboratories, Inc.). The microfluidic cells were designed in AutoCAD and 3D-printed with polylactic acid using an Ultimaker 3 (filament and printer from Ultimaker B.V., Netherlands). Cyclic voltammetry and impedance measurements was performed with a single junction Ag/AgCl/3 M KCl reference electrode (Model 6.0726.100, Metrohm Nordic AB) and a platinum rod (Model 6.0331.000, Metrohm Nordic AB) as the counter electrode. For the impedance measurements, a modified glassy carbon (GC) electrode tip (model 6.1204.300, Metrohm Nordic AB) was used as the working electrode, to mimic the actuator in the microfluidic cell.

Preparation of the actuators. The actuator for K-uptake in the potentiometric cell (**Figure 1c**, main manuscript) was prepared as follows. The counter and reference electrodes of commercially available screen-printed electrodes (Carbon, DRP-150, Dropsens, Metrohm Nordic) were covered by a mask made from adhesive transfer tape (3M 9471LE, 0.058 mm thick) and Mylar sheets (75 μm thick, RS Components, Sweden). In more detail, the mask was prepared using a Silhouette Cameo cutter (Silhouette Inc., USA) to cut a 4.2 mm diameter hole in the mylar sheet (slightly bigger than the diameter of the working electrode). Additionally, the Silhouette Cameo cutter was utilized to cut the adhesive transfer tape in the shape of a ring,

allowing the attachment of the mylar sheet to the substrate, without the adhesive touching any of the electrodes. Importantly, with the mask attached to protect the counter and reference electrodes, the working electrode was modified by depositing the CNTs through drop-casting 10x5 μL of 1 mg/mL CNT-suspension (2 min of drying between each addition). Subsequently, the ion-selective membrane was deposited by spin-coating, applying 20 μL of membrane cocktail (see **Table S1** for compositions) to the screen-printed electrode at 1500 rpm for 60 s (GreatCell Solar, Australia). Finally, the mask was removed, and the actuator inserted into the corresponding microfluidic cell.

For the optical measurements, the actuator was prepared in a very similar way, but with the following adjustments: i) a transparent gold electrode was used instead of carbon (Au, AUTR10, Dropsens, Metrohm Nordic), and ii) an additional air-bubble was supplementary blown in the center of the transparent gold electrode for each deposition of CNTs, using the micropipette, thus pushing the CNT-suspension to the side and providing a free path for the transmission of light through the optical microfluidic cell (ca 1 mm wide opening in the deposited CNTs layer).

Preparation of the sensors. The potentiometric sensor was based on screen-printed carbon electrode (Carbon, DRP-150, Dropsens, Metrohm Nordic), and a multi-layered mask governing the thickness of the ion-selective membrane. The mask was made from multiple layers of mylar sheets (125 μm and 75 μm thick, RS Components, Sweden) and adhesive transfer tape (3M 9471LE, 0.058 mm thick) to achieve a total thickness of 450 μm. Each mylar sheet and transfer tape were cut in the shape of a ring with an outer diameter of 8 mm and an inner diameter of 4 mm (size of working electrode) using the Silhouette Cameo Cutter. Then, 10x2 μL 1 mg/mL CNT-suspension was drop casted onto the working electrode with 2 min of drying between each addition. Subsequently, the membrane was drop casted onto the electrode in additions of 5 μL at a time (10x5 μL, see **Table S2** for compositions), attaining a ca. 450 μm thick membrane. The electrode was then left to dry for 5 hours and finally conditioned overnight in 10 mM KCl for the K-sensor, or 10 mM NaCl for the Na-sensor.

The K-selective optode was prepared by means of spin coating the K-optode cocktail, with a composition analogous to a previous work (**Table S2**),[2] at 1500 rpm for 60 s onto a 10x10 mm glass-slide.

2. Additional Electrochemical Experiments

Influence of the ETH500 present in the membrane. **Figure S6a** displays the Nyquist plots of membranes with and without ETH500 at the OCP in 10 mM KCl solution. The diameter of the semicircle displayed in the plots, which represents the bulk resistance of the membrane,[3] was found to decrease with the presence of ETH500 in the membrane. The same trend is also observed at –0.4 V vs OCP (**Figure S6b**), but with a slight increase in the resistance compared with that observed at OCP conditions (**Figure S6a**).

The influence of ETH500 in the actuators' electrochemical behavior was further studied by cyclic voltammetry at different scan rates (5–500 mV/s) in 1 mM KCl / 10 mM $MgCl_2$ solution as supporting electrolyte. **Figure S7a** shows the voltammograms of the actuator with ETH500, presenting no peaks. Instead, a capacitive region can be observed from –0.1 to –0.4 V (with respect to the initial OCP), where a rather constant current was observed for all scan rates. Then, as the potential sweep reverses towards positive potentials, a more resistive behavior can be observed, suggesting a faster charge transfer during ion-uptake, compared to ion-release. **Figure S7b** shows the voltammogram of the actuator without ETH500, presenting a fully resistive behavior, which confirms the increase in resistance observed in the impedance measurements presented in **Figure S6**. The electrochemical behavior was further studied inside the thin layer cell (**Figure 1c** in the main manuscript) in 1 mM KCl / 10 mM $MgCl_2$ solution as the supporting electrolyte. The results are presented in **Figure S7c**. A resistive behavior is observed from 50 mV/s and at higher scan rates, likely due to the increase in resistance in the thin-layer compartment for the solution.[4] Finally, the need for a supporting electrolyte in potential sweep experiments was verified by cyclic voltammetry inside the thin layer cell, where the resistive behavior increases upon the removal of the supporting electrolyte (**Figure S7d**).

Impedance on the different CNTs. Figure S12 displays the impedance spectra of electrodes made of different CNTs in 0.1 M KCl, all presenting close to the expected 90° capacitive lines.[5] By considering the imaginary impedance at the lowest frequency (10 mHz), the capacitance of the CNTs can be calculated to be 71, 10262 and 2010 $\mu F/cm^2$ for the COOH-CNTs, ODA-CNTs and CNTs, respectively.

3. Calculations of Diffusion Profiles in the Thin Layer

To estimate the time needed to deplete the concentration in the thin-layer sample from 1 to 0.1 mM of K^+, a finite differential step model was established in Matlab using a simplified approach to that published by Morf and co-workers.[6] An illustration of the considered spatial-domain is presented in **Figure S4a**. In more detail, the model considers the actuator (positioned at $x = 0\ \mu m$) in contact with a 1-dimensional thin-layer sample with the sensor positioned at $x = 50\ \mu m$. Each finite step is positioned with 1 μm of distance between them ($\Delta x = 1\ \mu m$). A fast charge transfer at the actuator-water interface is assumed and hence, it is treated as instantaneous (i.e., constant K^+ concentration at the actuator interface for $t > 0$, $x = 0\ \mu m$), and only diffusion is considered in the aqueous phase (spatial domain; $0\ \mu m < x < 50\ \mu m$) when estimating the time of uptake by the actuator (i.e., no migration effects). Furthermore, as the peristaltic pump is turned off during the ion-uptake, flow is not expected to contribute to mass-transport. Before the activation of the actuator, the concentration $c(x,t) = 1\ mM$ is constant inside the thin-layer sample ($t = 0$, **Figure S4b**). Then, upon activating the potential, the concentration at the actuator-solution interface changes instantly according to equation S1, granted the assumptions of the model

$$c(x = 0, t) = 0.1, t > 0 \quad \text{(S1)}$$

Then, for each time increment ($\Delta t = 0.01\ ms$) from 0 to 10 s, the concentration profile inside the thin-layer solution (i.e., for each finite spatial element) is calculated according to equation S2:

$$c(x, t + \Delta t) = c(x,t) + \big(c(x - \Delta x, t) - 2c(x,t) + c(x + \Delta x, t)\big)\frac{D_{K^+,aqeous}}{\Delta x^2}\Delta t \quad \text{(S2)}$$

where $D_{K^+,aqeous}$ equals 1.84x10^{-5} cm^2/s,[7] being the diffusion coefficient of K^+ in water.

At $x = 50\mu m$ (i.e., the solution-sensor interface), no more spatial elements can be considered for $x > 50$, and equation S3 is applied instead, considering the reflection at the mentioned interface.

$$c(50\ \mu m, t + \Delta t) = c(50\ \mu m, t) + 2\big(c(x,t) - c(x - \Delta x, t)\big)\frac{D_{K^+,aqeous}}{\Delta x^2}\Delta t \quad \text{(S3)}$$

Finally, the resulting concentration profiles inside the thin-layer solution at different times after the activation of the actuator are presented in **Figure S4b**, and the corresponding dynamic concentration at the sensor ($x = 50\ \mu m$) is presented in **Figure S4c**. As a result of the fast mass transport in thin-layer domains, the calculations suggest that if the charge transfer at the actuator is fast, it is possible to exhaustively deplete the K^+ in the sample within 3 seconds, being reasonably close to the results observed in **Figure 2** in the main manuscript (13 s), where any discrepancies between simulations and results can be ascribed to the assumptions made (i.e., instantaneous charge transfer, no migration effects, 1D-diffusion profile, sensor-response time).

4. Tables

Table S1. Composition of the membranes used to tailor the actuators. All membrane cocktails additionally contained a 2:1 weight ratio between the plasticizer (DOS) and PVC. K-L=Valinomycin.

Experiment	Membrane	Composition (mmol/kg membrane)
Normal actuator	K-L<br>NaTFPB<br>ETH500	(68)<br>(35)<br>(35)
Control experiment without ionophore	NaTFPB<br>ETH500	(37)<br>(38)
Control experiment without ETH500	K-L<br>NaTFPB	(71)<br>(36)
Control experiment without cation exchanger	K-L<br>ETH500	(68)<br>(35)

Table S2. Composition of the membranes used to fabricate the sensors. All membrane-cocktails additionally contained a 2:1 weight ratio between the plasticizer (DOS) and PVC. K-L=Valinomycin, Na-L=Sodium Ionophore X, H-L=Chromoionophore I.

Sensor / Experiment	Membrane	Composition (mmol/kg membrane)
K-Sensor / Potentiometric Measurements	K-L<br>NaTFPB<br>ETH500	(68)<br>(35)<br>(35)
Na-Sensor / Potentiometric Measurements	Na-L<br>NaTFPB	(73)<br>(37)
K-Optode / Optical Measurements	K-L<br>H-L<br>NaTFPB	(18)<br>(16)<br>(17)

5. Figures

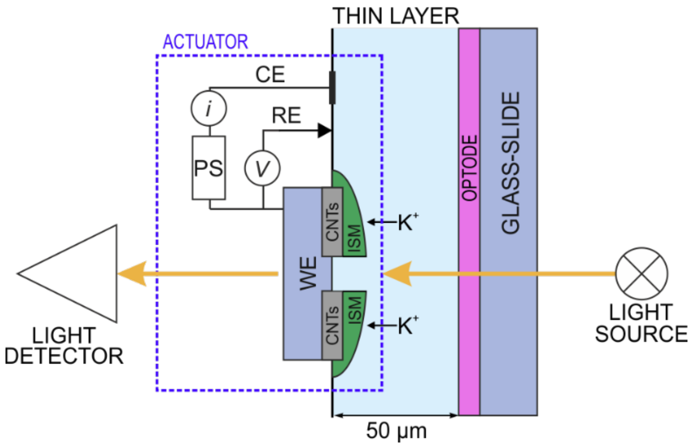

Figure S1. Scheme of the experimental setup where the sensor is an optical one. ISM=Ion-selective membrane; CNTs=Carbon nanotubes; WE=transparent working electrode (Au); CE=counter electrode; RE=reference electrode; PS=power supply (potentiostat); Light Source=Deuterium- and Halogen-lamp.

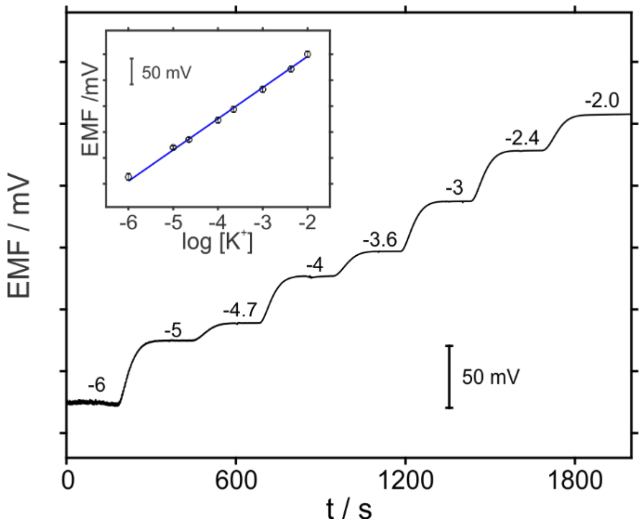

Figure S2. The dynamic potentiometric response of the potassium potentiometric sensor at increasing potassium concentrations in the thin layer cell. Inset: the corresponding calibration graph (n = 3) with error bars representing the standard deviation.

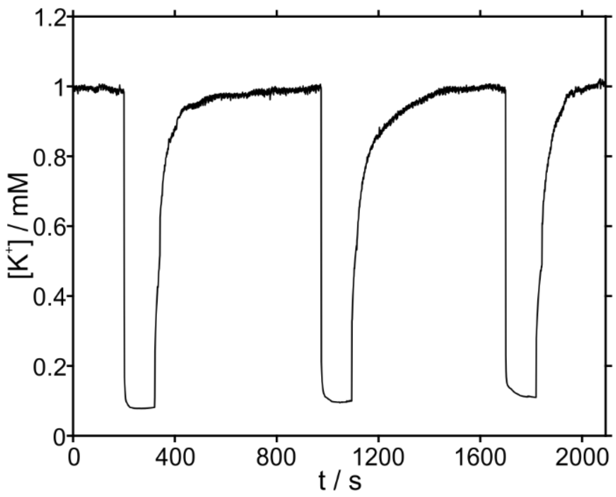

Figure S3. The concentration-time profile for three consecutive uptakes in 1 mM KCl solution through activation of the actuator (–0.4 V with respect to OCP for 120 s). 30 s after each uptake was finished, the peristaltic pump was turned on to regenerate the baseline for the next uptake.

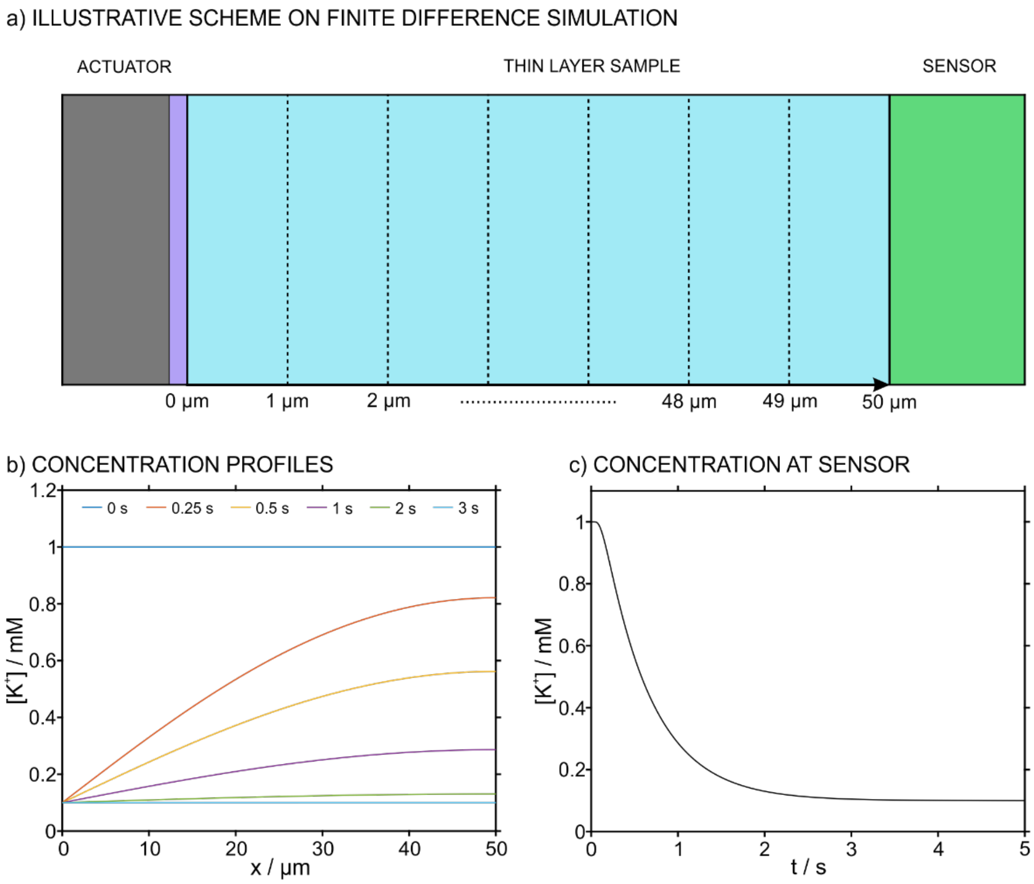

Figure S4. a) Scheme for the finite spatial elements considered for the calculation of the concentration profiles. b) The concentration profiles inside the thin-layer sample at different times where the sensor is located at x=50 μm and the actuator at x=0 μm. c) The concentration profile at the indicator surface versus time.

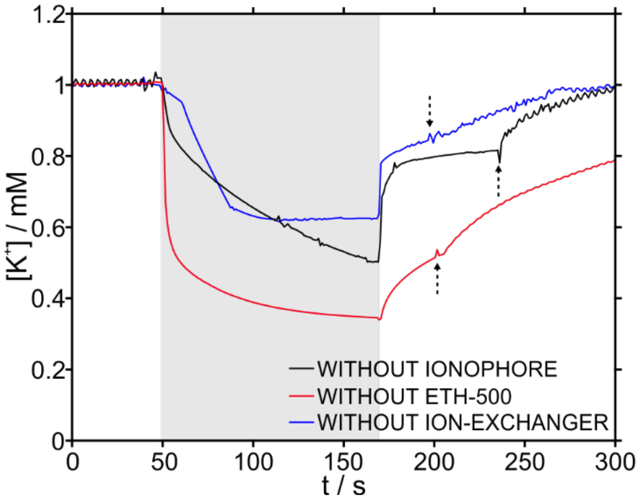

Figure S5. The concentration-time profiles for different membrane compositions in the actuator in 1 mM KCl solution and applying –0.4 V with respect to OCP for 120 s. The arrows indicate when the peristaltic pump was turned on to regenerate the baseline for the initiation of a new experiment.

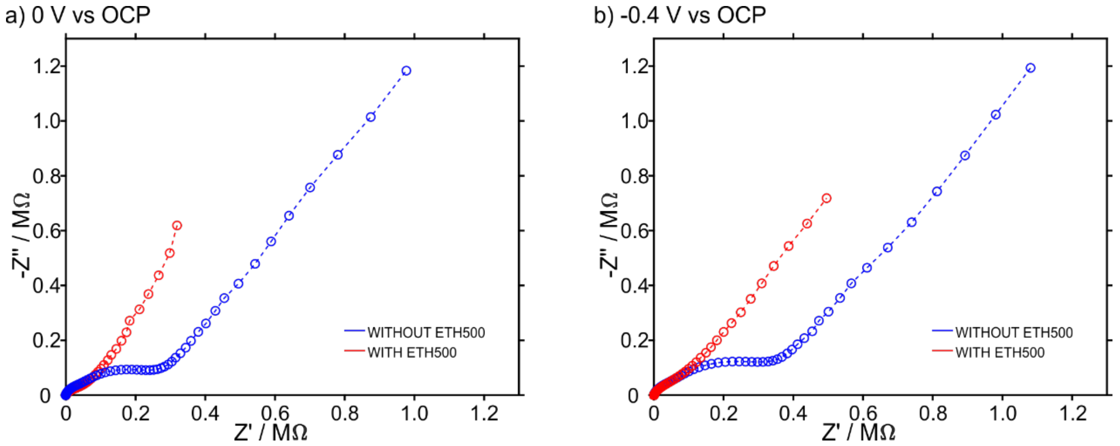

Figure S6. Electrochemical impedance spectra GC/ISM with and without ETH500, at a) E_{DC} = 0 V vs OCP and b) at E_{DC} = -0.4 V vs OCP. Experiments were performed in 0.1 M KCl, in the frequency range 100 kHz–10 mHz with ΔE_{AC} = 10 mV.

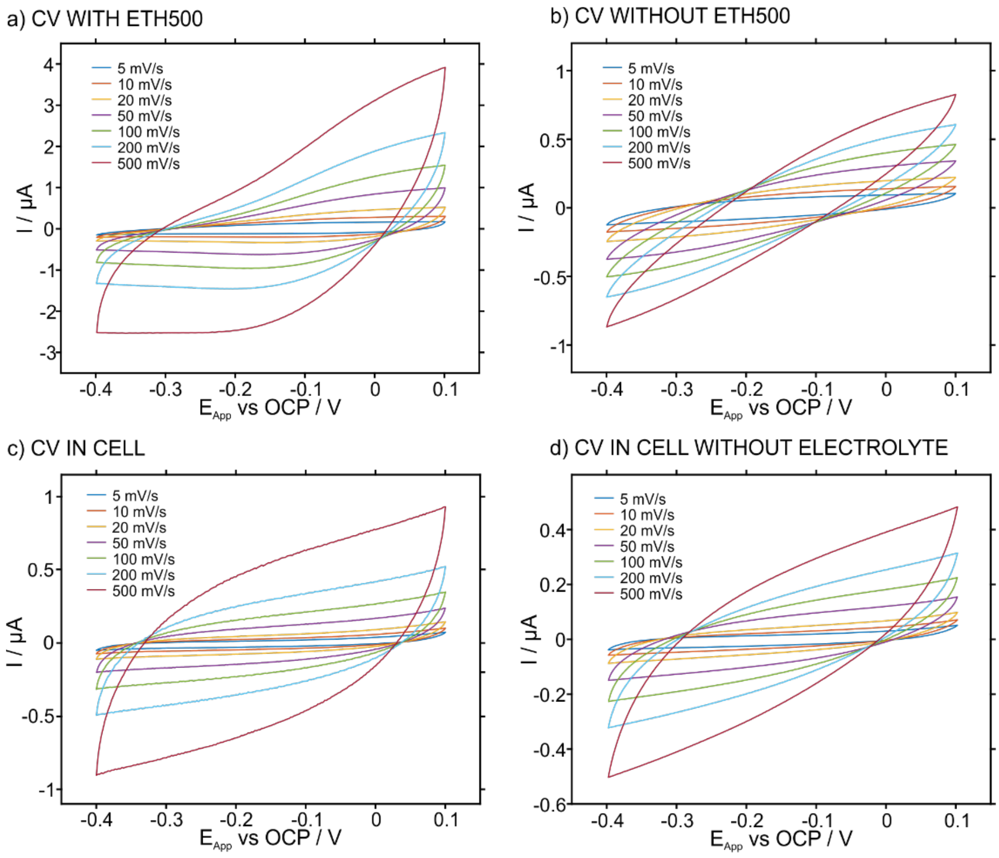

Figure S7. Cyclic voltammograms (CV) at increasing scan rates (5–500 mV/s) for a) a membrane with ETH500, and b) a membrane without ETH500, performed in the beaker. c) CV using a membrane with ETH500, performed in the microfluidic cell. d) CV of a membrane with ETH500, performed in the microfluidic cell without background electrolyte. Experiments were performed in 1 mM KCl / 10 mM $MgCl_2$ background electrolyte to decrease the solution resistance (except for **Figure S1d**). Sweeps were conducted between –0.4 and 0.1 V vs OCP (ca 0.1 V vs the reference electrode).

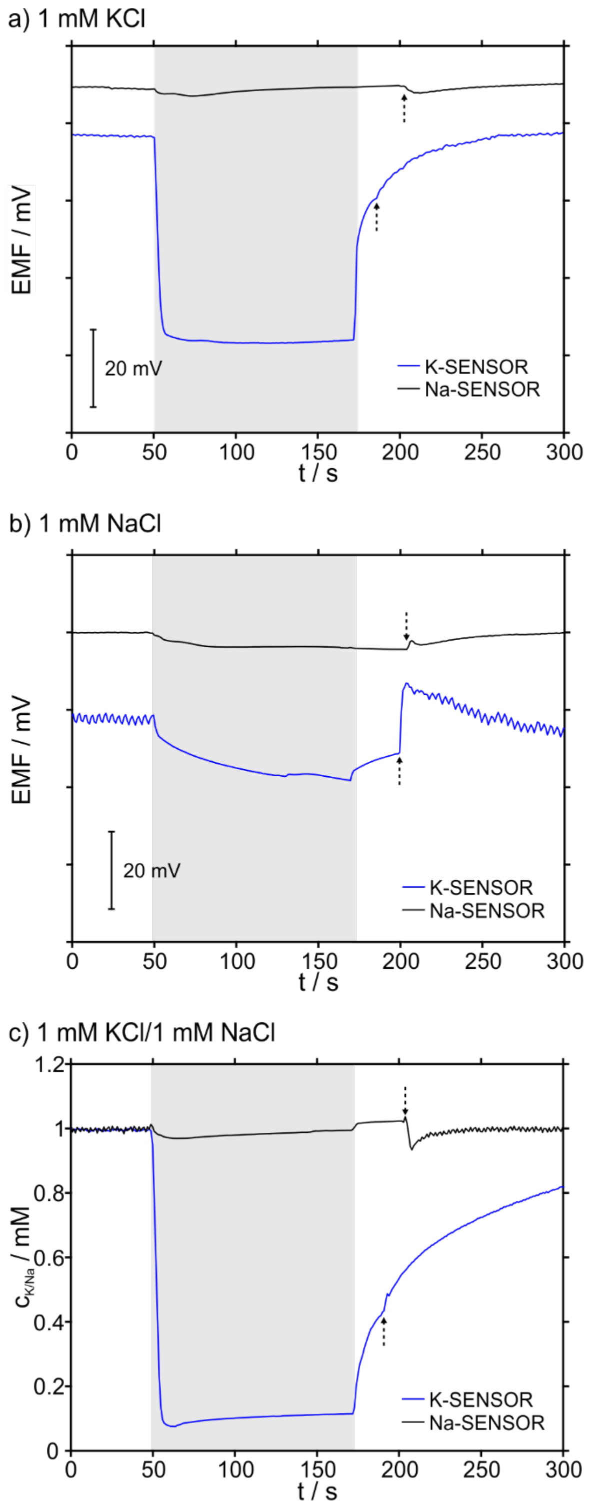

Figure S8. The EMF-time profiles of a K potentiometric sensor and a Na potentiometric sensor in a) 1 mM KCl, b) in 1 mM NaCl and c) 1 mM KCl/1mM NaCl solution.

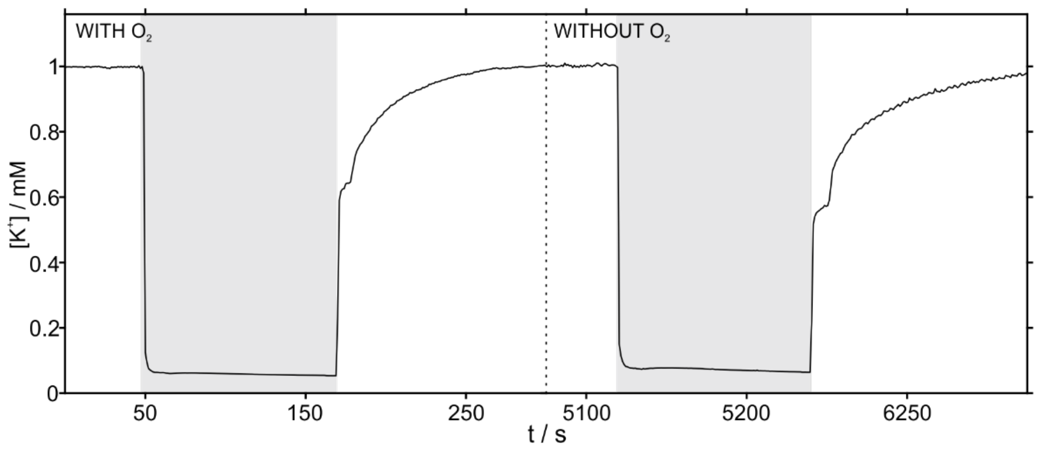

Figure S9. The concentration-time profile for the K-uptake with and without O_2 in 1 mM KCl solution through activation of the actuator (–0.4 V with respect to OCP for 120 s). 30 s after each uptake was finished, the peristaltic pump was turned on to regenerate the baseline for the next uptake. The dotted line represents a cut in time during the purging of the sample with N_2.

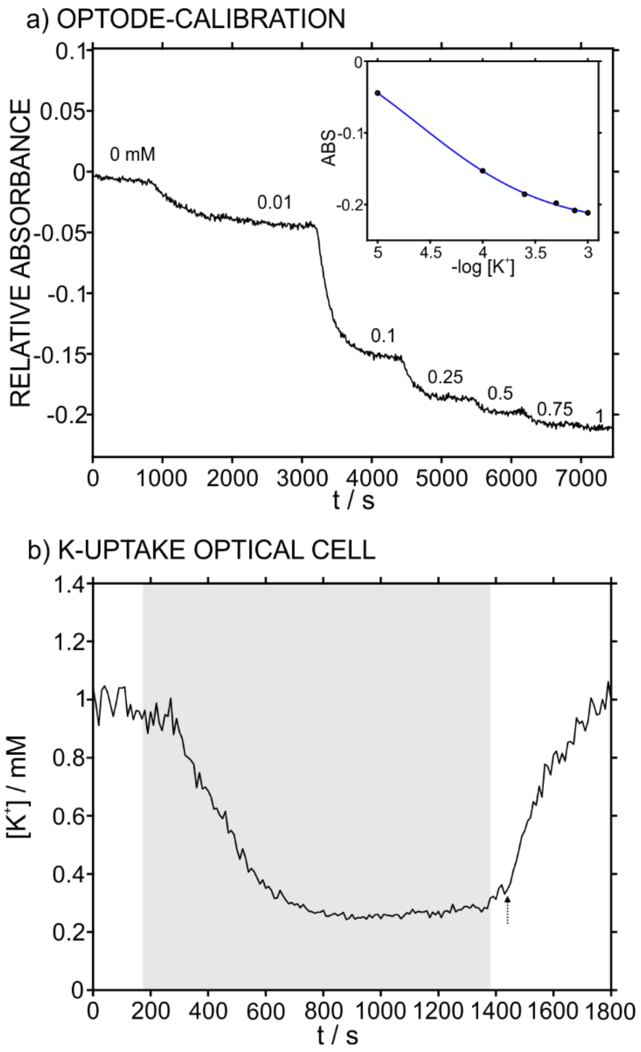

Figure S10. Optical experiments with the potassium optode, where a) presents the relative absorbance versus time for the optode calibration (inset: the corresponding calibration graph with a sigmoidal fit) and b) the monitored uptake from the actuator with the optical readout, with an applied potential of –700 mV (with respect to OCP) for 1200 s (gray area). The arrow indicates when the peristaltic pump was turned on to regenerate the base line. Background=10 mM TRIS-HCl, pH 7.5.

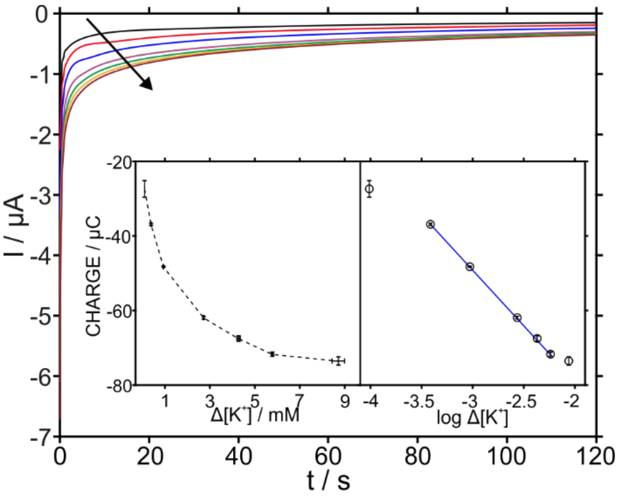

Figure S11. The current-time profiles for different KCl concentrations, applying –0.4 V (with respect to the OCP) for 120 s (black curve 0.1 mM, red 0.4 mM, blue 1 mM, purple 3 mM, green 5 mM, yellow 7 mM and dark red 10 mM). The arrow displays the trend with increasing KCl concentrations. Inset (left): correlation between generated charge and measured concentration difference, with error bars corresponding to one standard deviation (n=3). Inset (right): the correlation between generated charge and the logarithm of the total concentration difference, blue line presenting the linear fit ($Q = -137.6 - 29.5 \times \log[K^+]$, R^2=0.9999).

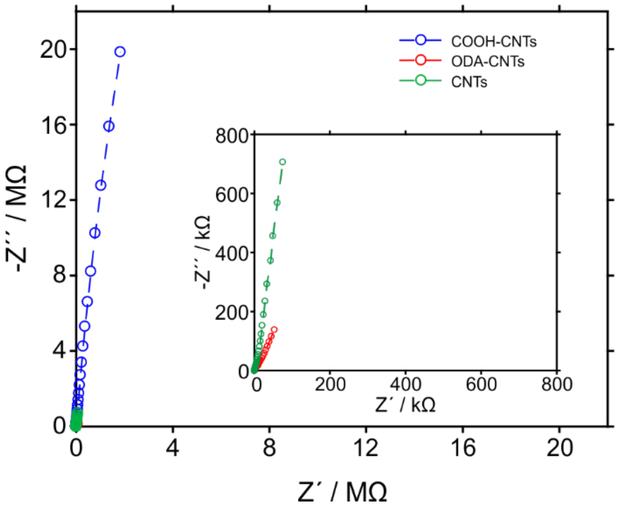

Figure S12. Electrochemical impedance spectra observed for the different types of CNTs. Inset: magnification on the impedance spectra of the ODA-CNTs and CNTs. Data was acquired in 0.1 M KCl, in the frequency range 100 kHz–10 mHz with ΔE_{AC} = 10 mV.

References

1. D. Yuan, A. H. C. A., M. Ghahraman Afshar, N. Pankratova, M. Cuartero, G.A. Crespo, E. Bakker, All-Solid-State Potentiometric Sensors with a Multiwalled Carbon Nanotube Inner Transducing Layer for Anion Detection in Environmental Samples. *Analytical Chemistry* **2015,** *87* (17), 8640−8645.
2. K. Wang, K. S., W.E. Morf, U.E. Spichiger, W. Simon, E. Lindner, E. Pungor, Characterization of Potassium-Selective Optode Membranes Based on Neutral lonophores and Application in Human Blood Plasma. *Analytical Sciences* **1990,** *6* (5), 715-720.
3. T. Han, U. V., J. Bobacka, Influence of Electrode Geometry on the Response of SolidContact Ion-Selective Electrodes when Utilizing a New Coulometric Signal Readout Method. *ChemElectroChem* **2016,** *3*, 2071-2077.
4. I.B. Goldberg, A. J. B., Resistive effects in thin electrochemical cells: Digital simulations of current and potential steps in thin layer electrochemical cells. *Journal of Electroanalytical Chemistry and Interfacial Electrochemistry* **1972,** *38* (2), 313-322.
5. G.A. Crespo, S. M., J. Bobacka, F.X. Rius, Transduction Mechanism of Carbon Nanotubes in Solid-Contact Ion-Selective Electrodes. *Analytical Chemistry* **2009,** *81*, 676–681.
6. W.E. Morf, E. P., N.F. De Rooij, Computer simulation of ion-selective membrane electrodes and related systems by finite-difference procedures. *Journal of Electroanalytical Chemistry* **2007,** *602* (1), 43-54.
7. Gosting, L. J., A Study of the Diffusion of Potassium Chloride in Water at 25° with the Gouy Interference Method. *Journal of American Chemical Society* **1950,** *72* (10), 4418–4422.
